# Sequential PBM–Saffron Treatment in an Animal Model of Retinal Degeneration

**DOI:** 10.3390/medicina57101059

**Published:** 2021-10-03

**Authors:** Mattia Di Paolo

**Affiliations:** 1 Department of Pharmacy, University of Pisa, Via Bonanno, 6, 56121 Pisa, Italy; m.dipaolo@bio-aurum.it; 2 Interuniversity Consortium Biostructures and Biosystems National Institute, Via Medaglie d’Oro 305, 00136 Roma, Italy; 3 Department of Biotechnological and Applied Clinical Sciences, University of l’Aquila, Via Vetoio, 1, 67100 l’Aquila, Italy; 4 Bio Aurum srl, Via Mangionello, 12, 73024 Maglie, Italy

**Keywords:** retinal degeneration, oxidative stress, photobiomodulation, saffron, neuroprotection, synergistic effect

## Abstract

*Background and Objectives:* Saffron treatment and photobiomodulation (PBM) are non-invasive therapeutic approaches able to mitigate and stabilize retinal degenerative diseases such as age-related macular degeneration (AMD). Although different, these therapies partially match their modulated pattern of genes. Recent attempts to find an additive effect by coadministration of saffron and PBM have failed. Instead, in this study, a different protocol to increase neuroprotection by providing consecutive saffron and PBM treatment administration is suggested. *Materials and Methods:* Albino rats, whose retinal damage was caused by light exposure (LD, light damage), were subjected to differential treatment protocols before and after LD: (1) PBM followed by saffron; and (2) single treatments of PBM. Thinning of the photoreceptor layer and neuro-inflammatory markers for gliosis and microglia were assessed via immune-histochemical techniques. *Results:* Results confirm that PBM and saffron alone cope with retinal neurodegenerative processes, preserving retinal thickness and gliosis and microglia invasion in a differential way. However, the synergistic effect of the combined treatment was restricted to the early neuroinflammation, even when provided sequentially. *Conclusion:* The broad spectra of action of both neuroprotectants require further investigation to identify other key pathways helpful in enhancing the effects of these two approaches in combination.

## 1. Introduction

Age-related macular degeneration (AMD) is a multifactorial retinal neurodegenerative disease in which aging, genetic polymorphisms, and environmental factors lead to an irreversible lack of visual perception by activating neuro-inflammatory and neurodegenerative processes [1]. Several therapies have been developed to reduce the progression of the disease. In particular, significant results have been obtained by reducing environmental risks such as smoking, by providing the AREDS (Age-Related Eye Disease Study) formulation [2,3], or with intravitreal injection of antibodies such as those against vascular endothelial growth factor (anti-VEGF) [4].

Photobiomodulation (PBM) offers a suitable approach to treat retinal neurodegeneration. This non-invasive therapy consists of a 670 nm light treatment to enhance cytochrome C oxidase activity and, through a complex pathway, mitigate the neuroinflammation [5,6,7]. In particular, PBM reduces complement propagation, stress-related markers in Muller cells [7,8], and lipid peroxidation [5], alongside modulating the cellular transcriptome. Furthermore, it was shown that PBM could reduce pathological drusen and preserve visual perception in patients with AMD [9,10].

Similar results have been obtained upon saffron treatment [11]. The neuroprotective effect of this spice administered as a diet supplementation has already been confirmed by several clinical trials [12,13,14,15,16,17]. Specific mechanisms of saffron neuroprotection are still under investigation [11]. However, several studies suggest that saffron is involved in cellular transcriptome modulation [18,19], metalloproteinase activity regulation [20], and purinergic and cannabinoid receptor expression [21,22], as well as mitochondrial activity [23].

The microarray study of Natoli et al. [18] suggests a partial match in the genes modulated by PBM and saffron treatment, respectively. Furthermore, previous works aimed to characterize and compare their neuroprotective mechanisms to enhance their effectiveness [8,23]. Surprisingly, in vivo experiments showed no additive effect in the simultaneous application of PBM and saffron, suggesting a competition in the activation of the shared pathways and possible negative interference between them [23]. In this study, we extended those previous results [23] in order to understand whether a different administration protocol might overcome this negative interaction. Using the same animal model of retinal neurodegeneration of the previous study—light-damaged albino rats—we applied both treatments with the sequential criteria of before or after the light exposure (light damage (LD)). According to a previous study [8], where in comparison with prolonged saffron treatment, prolonged PBM exposure is less effective, we selected a combined protocol with short PBM exposure preceding the LD followed by the saffron treatment.

## 2. Materials and Methods

All the experiments were conducted following the ARVO Statement for the Use of Animals in Ophthalmic Research and authorized by the Ministry of Health (authorization number 83/96-A of 29 November1996). Sprague Dawley rats were fed ad libitum (4RF18, Mucedola srl, Milan, Italy) and bred at 5 lux with dark:light cycles of 12:12 h. Animals were organized in 5 experimental groups, 5 rats each. The LD control group were exposed for 24 h at 1000 lux; the PBM + LD group was PBM treated for 7 days, before the LD; the LD + PBM group was conditioned with PBM for 7 days following the LD; the PBM + LD + saffron group was treated with PBM for 7 days before the LD, and subsequently rats were exposed with saffron for 7 days following the LD. The healthy control group did not receive any treatment. All the rats were euthanized 7 days after the LD.

### 2.1. PBM and Saffron Treatments

PBM treatments were manually applied for 7 days before and 7 days after the LD. Rats were treated daily in small cages, for 3 min. The WARP 75 source (610–730 nm, peak at 670 nm. Quantum Devices Inc., Barneveld, WI, USA), placed 2.5 cm away from the animal, provided 4.0–4.5 J/cm^2^ at the eye level. Rats were allowed to move freely in the cage and the exposure was only performed when their eyes faced towards the enclosure and at the right distance. The PBM exposure provided was a non-toxic treatment [18,24]. The saffron treatment consisted of an aqueous extract of saffron provided as a daily diet supplementation (Saffron REPRON, patent: W02015/145316) (1 mg/kg/day). In particular, animals were weighed every week to rectify the amount of saffron required, and a proper daily volume of saffron extract was identified by previous tests and dispensed in an additional feeding bottle (additional to the water bottle). The spontaneous preference for the extract of saffron to the water guaranteed a correct and continuous uptake of the spice. The saffron dosage used is a completely safe treatment [25,26].

### 2.2. Tissue Processing

Explanted eyes were immediately placed in 4% paraformaldehyde buffer solution for 6 h. Subsequently, the cryoprotection was provided via rinses with graded concentrations of sucrose solutions. The eyes were embedded in mounting medium (Tissue Tek OCT compound; Sakura Finetek, Torrance, CA, USA) and frozen via liquid nitrogen immersion. Cryo-sections were collected on coated gelatin-polylysine by using a cryostat (CM1850 Cryostat; Leica, Wetzlar, Germany) and stored at −20 °C.

### 2.3. Immunohistochemistry

Retinal cryo-sections were rinsed with physiological saline buffer (PBS, 003002, Thermofisher, Waltham, MA, USA) and exposed to a blocking buffer (10% bovine serum albumin (BSA, A9647, Merk, Darmstadt, Germany)) for 20 min. Afterwards, retinae were left overnight at 4 °C with primary antibodies (1:500 ionized Ca^2+^-binding adapter molecule-1, Iba1, 019-19741, Wako, Osaka, Japan; 1:1000 Glial fibrillary acidic protein, GFAP, Z0334, Dako, Santa Clara, CA, USA). Sections were washed with PBS and incubated with the corresponding secondary antibodies (1:200 ALEXA Fluor; Molecular Probes, Invitrogen Carlsbad, CA, USA) for 2 h at 37 °C. The dye nucleus staining preceded the coverslip application. Images of retinae were acquired by a confocal microscope (Nikon, Tokyo, Japan) and analyzed with ImageJ software. GFAP expression by the Muller cells and outer nuclei layer (ONL) thickness were measured according to the protocol from a previous study [23]. Statistical tests were performed with Prism7 (GraphPad Software, San Diego, CA, USA). Iba1-positive cells were manually counted with a fluorescence microscope (Nikon, Tokyo, Japan).

## 3. Results

### 3.1. Morphological Analysis: PBM Preconditioning Is Enough to Preserve Photoreceptors Layer

**Figure 1:**
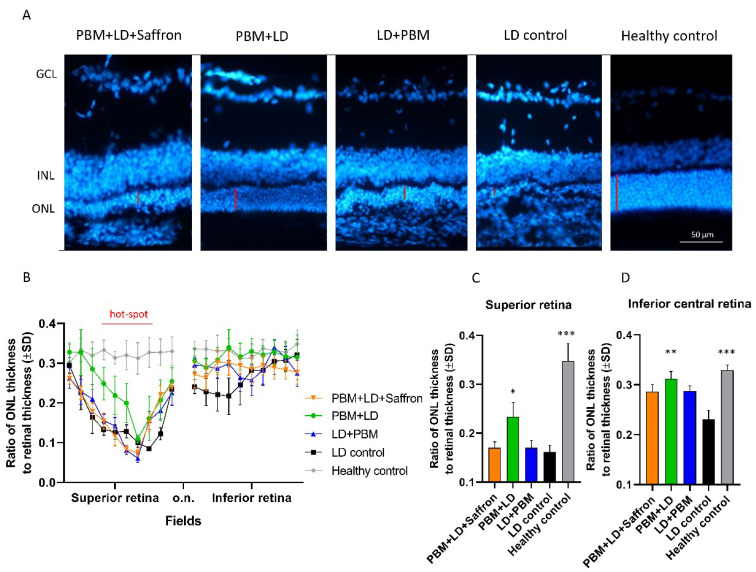
Saffron and photobiomodulation effect on the ONL retinal thickness. (**A**) Representative pictures of nuclei-stained layers (DAPI) of the superior retina. Red lines on the pictures indicate the ONL thickness. (**B**) The graph shows the mean ONL thickness along the retina, from the superior to the inferior side, following the axes through the optic nerve (o.n.). The light-induced thinning mostly involves the superior retina, whose area is identified as the “hot-spot”. (**C**,**D**) Histograms describe the ratio of the ONL thickness to retinal thickness in the superior and inferior central retina, respectively. All applied treatments tend to preserve retinal thickness in less damaged areas, neighboring the hot-spot, such as the inferior central retina (5 fields from the o.n.) (**D**). However, the PBM + LD treatment has a statistical significance for the neuroprotective effect in the inferior central retina and superior retina (**C**). Statistical tests: Kruskal–Wallis followed by Dunn’s test. Superior retina: PBM + LD + saffron vs. LD control *p* > 0.05; PBM + LD vs. LD control *p* = 0.0311; LD + PBM vs. LD control *p* > 0.05; healthy control vs. LD control *p* < 0.001; PBM + LD + saffron vs. PBM + LD *p* > 0.05. Inferior central retina: PBM + LD + saffron vs. LD control *p* > 0.05; PBM + LD vs. LD control *p* = 0.0058; LD + PBM vs. LD control *p* > 0.05; healthy control vs. LD control *p* < 0.001; n = 5. Significance indicator: *** *p* < 0.001; ** *p* < 0.01; * *p* < 0.05.

High amounts of light exposure in albino rats induce a specific pattern of retinal neurodegeneration, which starts in the superior side of the retina (known as the hot-spot area) and spreads out over time [27,28]. After seven days post light exposure, rats in the LD control group had a severe thinning of the ONL. Figure 1A shows superior retinae sections with nuclei staining from all experimental groups; the ONL thickness related to the treatment is underlined in red. Figure 1B shows the mean ONL thickness along the retina, measured from the superior to the inferior side, following the axes through the optic nerve (o.n.). The PBM + LD group (green line) demonstrates a greater neuroprotective effect, which involves both the peripheral-superior retina and the inferior retina. The other treatment protocols show a small effect on less damaged tissue near the hot-spot area, such as in the inferior central retina. Statistical tests: Kruskal–Wallis followed by Dunn’s test. Superior retina: PBM + LD + saffron vs. LD control *p* > 0.05; PBM + LD vs. LD control *p* = 0.0311; LD + PBM vs. LD control *p* > 0.05; healthy control vs. LD control *p* < 0.001. Inferior central retina: PBM + LD + saffron vs. LD control *p* > 0.05; PBM + LD vs. LD control *p* = 0.0058; LD + PBM vs. LD control *p* > 0.05; healthy control vs. LD control *p* < 0.001).

### 3.2. Analysis of Gliosis: Combination of PBM and Saffron Markedly Reduces Early Neuroinflammation

**Figure 2:**
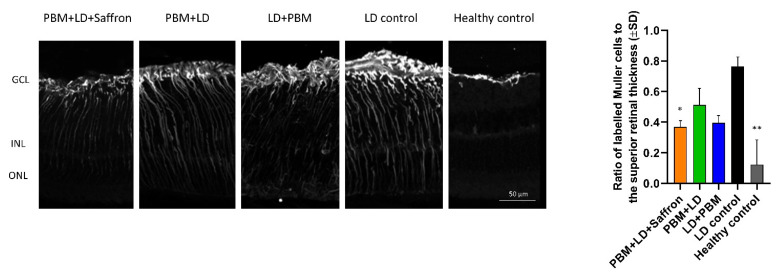
Impact of the treatments on gliosis. Representative pictures of the retinae sections immuno-labeled for GFAP. The relative histogram to the right shows a significant reduction in GFAP expression in all treatments. In particular, the labeling of the Muller cells’ branches appears less extended across the retina. Statistical tests: Kruskal–Wallis followed by Dunn’s test. PBM + LD + saffron vs. LD control *p* = 0.0348; PBM + LD vs. LD control *p* > 0.05; LD + PBM vs. LD control *p* > 0.05; healthy control vs. LD control *p* < 0.01; PBM + LD + saffron vs. PBM + LD *p* > 0.05; n = 5. Significance indicator: ** *p* < 0.01; * *p* < 0.05.

LD produces acute tissue damage in which neuro-inflammation is mostly involved in the early neurodegenerative processes [29]. Muller cells are retinal glial cells extending across the entire retina, which play an important role as a retinal stress reporter. In particular, high light exposure induces Muller cells to increase glial fibrillary acidic protein (GFAP) expression on their cellular membrane [30]. Figure 2 describes the GFAP expression in the retinal sections of each experimental group. Labeled branches of Muller cells in all treated retinae are in summary shorter than the LD control group. (Histogram in Figure 2. Statistical tests: Kruskal–Wallis followed by Dunn’s test. PBM + LD + saffron vs. LD control *p* = 0.0348; PBM + LD vs. LD control *p* > 0.05; LD + PBM vs. LD control *p* > 0.05; healthy control vs. LD control *p* < 0.01; PBM + LD + affron vs. PBM + LD *p* > 0.05).

### 3.3. Microglia Activation: PBM Preconditioning of the Retina Mitigates Iba1 Positive Cells Invasion

**Figure 3:**
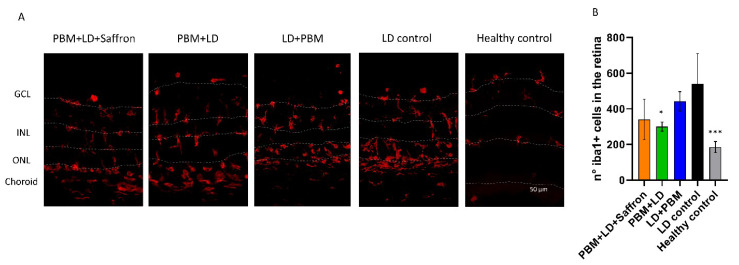
Effect of saffron and photobiomodulation on microglial activation in the retina. (**A**) Representative pictures of transversal sections of the superior retinae immuno-labeled to Iba1. Dashed lines underline borders among the retinal layers. (**B**) Counts of microglial cells of the entire retinal section in each experimental group. The number of Iba1-positive cells is significantly lower compared to the LD group in all treatment protocols, except for the LD + PBM group. Statistical tests: Kruskal–Wallis followed by Dunn’s test. PBM + LD + saffron vs. LD control ns; PBM + LD vs. LD control *p* = 0.0405; LD + PBM vs. LD control ns; healthy control vs. LD control *p* < 0.001; PBM + LD + saffron vs. PBM + LD ns; n = 5. Significance indicator: *** *p* < 0.001; * *p* < 0.05.

Activated microglia cells play a dual role in the degenerated retina [31]. As a result of high light exposure, there is an initial rapid inflammatory response, which tries to lead back to physiological homeostasis. However, if the toxic insult is chronic, activated microglia can overreact and contribute to the disruption of the retinal tissue. Figure 3 shows the distribution of labeled microglia in the retina strata of all experimental groups. In healthy conditions, quiescent microglia are restricted to the inner retina (GCL and INL). After LD, resident and non-resident cells are attracted to the outer retina by the neurodegenerative signals [28]. Figure 3 shows the counting of retinal microglia in each group per retinal section. Both the pretreatment with PBM and the PBM–saffron combined protocol can slightly mitigate the microglia activation. On the other hand, the LD + PBM treatment does not seem to influence microglia invasion. (Statistical tests: Kruskal–Wallis followed by Dunn’s test. PBM + LD + saffron vs. LD control ns; PBM + LD vs. LD control *p* = 0.0405; LD + PBM vs. LD control ns; healthy control vs. LD control *p* < 0.001; PBM + LD + saffron vs. PBM + LD ns).

## 4. Discussion

In this study, we aimed to identify a new neuroprotective strategy to treat light-induced retinal degeneration. In a previous study [23], we showed that a simultaneous application of saffron and PBM does not enhance their neuroprotective effect while, on the contrary, it seems that an adverse interaction occurs. PBM + LD and PBM + LD + saffron are effective in mitigating different consequences of the initial damage, confirming their intriguing properties in improving retina resiliency [32]. In particular, PBM + LD significantly preserved the ONL morphology and reduced microglia invasion, while PBM + LD + saffron slowed down the neuroinflammation assessed by GFAP expression by Muller cells. This is in line with previously published data [8], which demonstrated that saffron treatment reduces gliosis processes better than PBM treatment alone. Specifically, we confirmed that PBM exposure in neurodegeneration requires to be finely administrated, probably more shortly [33] in comparison with saffron, whose positive effect on Muller cells was also underlined in other glial-dependent retinal dystrophies, such as in Fischer rats [26]. Furthermore, we confirmed that PBM treatment barely mitigates neurodegenerative processes if provided immediately after LD [33]. However, compared to the single treatments with PBM (PBM + LD and LD + PBM), the combined conditioning of PBM + saffron does not provide an enhancement in neuroprotection. This treatment strategy confirms that saffron and PBM have an antagonistic effect, even when provided in a sequential manner. Molecular mechanisms involved in this interaction are still unknown. However, Corso et al. [22] found that saffron treatment might reduce intracellular calcium through the inhibition of purinergic receptors [1]. On the other hand, Golovynska at al. [34] show that the exposition of near-infrared light might affect biological structure different from cytochrome c oxidase. In particular, they describe how PBM increases intracellular calcium via the activation of NMDA receptors [35]. Therefore, treatments applied might compete in the intracellular calcium flux. Furthermore, PBM and saffron neuroprotection might be affected by the physio-pathological context, which requires a different balance among antioxidants, trophic factors, or anti-inflammatory molecules [26]. Accordingly, a biphasic dose–response related to low-level light therapy was suggested [33]. Specifically, an excess of PBM treatment might negate the beneficial effect of a lower dosage. As gene modulation of single treatments with saffron and PBM is partially matched [18], it would be possible that saffron in this specific protocol of treatment boosts the PBM neuroprotection over the threshold. As both neuroprotectants have a wide range of action, further investigations are required to identify other key pathways modulated by both treatments and to refine an efficient combination protocol.

## Data Availability

The experimental data that support the figures within this paper and other findings of this study are hosted at the University of l’Aquila, Department of Biotechnological and Applied Clinical Sciences and can be accessed by contacting the corresponding author. A patent “Compositions based on saffron for the prevention and/or treatment of degenerative eye disorders” covering the topic of this manuscript has been filed on 20 March 2015 (W02015/145316) and is owned by Hortus Novus srl. Silvia Bisti, Rita Maccarone and Maria Maggi are the inventors of the patent.
